# Does the Conversion of Household Registration Actually Improve the Happiness of Migrant Workers in China?

**DOI:** 10.3390/ijerph17082661

**Published:** 2020-04-13

**Authors:** Xin-hao Liu, Li-min Han, Bin Yuan

**Affiliations:** College of Management, Ocean University of China, Qingdao 266100, China; liuxinhao@stu.ouc.edu.cn (X.-h.L.); cnqdhlm@163.com (L.-m.H.)

**Keywords:** household registration, migrant worker, happiness, group difference analysis

## Abstract

Migrant workers are an important human resource for economic and social development. Considering the government’s goal of serving and improving people’s livelihoods, improving the happiness of migrant workers is necessary. This study investigates in-depth the impact of the conversion of household registration on migrant workers’ happiness, which is represented by a multi-dimensional comprehensive index based on the propensity matching score model and data from the China Migrants Dynamic Survey (CMDS) in 2017. Moreover, this study explores the different effects of conversion among the groups divided by the characteristics of migrant workers. The results show that from an overall perspective, although the conversion of household registration could improve the happiness of migrant workers, the degree of this improvement is minor. Further, the characteristics of the different groups, including age, educational background, contracted land, collective dividends, and income significantly affect the improvement of happiness. The conversion of household registration has obviously improved the happiness of migrant workers with low educational backgrounds, low income, and contracted land. Based on these findings, the government should take more targeted actions to improve the positive effects of household registration among different migrant worker groups due to the different characteristics in the process of household registration system reform.

## 1. Introduction

The United Nations Sustainable Development Solutions Network (SDSN) released the World Happiness Report in 2019. This report ranked 156 countries and regions according to their overall scores of per capita GDP, social support, healthy life expectancy, social freedom, generosity, and corruption. China ranked 93rd with a score of 5.191, which indicates that the happiness of Chinese citizens is generally low. This report means that although economic growth has been rapid since the economic reform and opening up of China, the growth of Chinese national happiness has stagnated or even declined [1]. Easterlin (2012) shows that during the 20 years from 1990 to 2010, Chinese residents’ per capita consumption increased by nearly four times. However, their happiness did not increase significantly, showing a “positive U-shape” trend [2]. As China’s economic development enters its “new normal” stage, national happiness has become an important indicator for measuring the overall level of economic and social development. Improving domestic happiness has thus become an essential task for the Chinese government.

According to the National Happiness Report released by the Southwestern University of Finance and Economics in 2014, Chinese citizens are generally happy. However, the happiness of farmers is noticeably lower than that of urban residents. Does this mean that the conversion of household registration can improve the happiness of Chinese farmers? In recent years, China has begun to engage in the construction of new-type “people-oriented” urbanization. Previously, China’s urbanization construction focused on increasing the urbanization rate of the resident population. This entailed “material-oriented” urbanization construction. The resident population involved in this process does not require a local household registration. Therefore, the reform of household registration has been slow. Because various policies only promote the movement of rural labor to the city and are not concerned with the conversion of household registration. The people-oriented new-type urbanization shows that China’s urbanization construction has changed direction and has begun to highlight the importance of “people” in urbanization construction and the need to protect the interests and rights of the people involved in the process of urbanization. This new-type urbanization construction aims to improve people’s satisfaction and happiness, not just numbers.

The beginning of China’s household registration system was the “Regulations on the Registration of Household Registration of the People’s Republic of China” promulgated by the Chinese government in 1958. This policy restricts the inflow of rural populations into cities and establishes specific measures. Welfare gaps, such as the social security underlying urban and rural household registrations, have begun to appear. After that, a series of household registration policies designated by the Chinese government further widened the gap between the welfare of urban and rural residents. For this reason, the welfare differences affected by household registration have gradually become an urgent problem to be solved in household registration reforms, which is also an inevitable task of new-type urbanization construction. As the main body of those engaging in the conversion of household registration includes farmers living in cities, the happiness of rural laborers working and living in cities has gradually attracted attention. This type of rural laborer is generally called a migrant worker. In 2018, the National Bureau of Statistics of China released the National Economic and Social Development Statistics Bulletin, which showed that the urbanization rate of the resident population in China reached 59.58%, but the urbanization rate of household registration was only 43.37%. The reason for this statistic is that there are a large number of migrant workers living in cities without urban household registration. In total, 71.8% of new urban residents are migrant workers with rural household registration. It can be seen that urbanization in China mainly involves urbanization of the population, not urbanization of household registrations [3]. Migrant workers are the basic force for promoting China’s economic and social development, but they are discriminated against in the urban labor market. Most of these workers are engaged in low-quality jobs, and their welfare cannot be guaranteed. Moreover, the phenomenon of different pay for equal work often occurs [4]. Migrant work is an essential part of urban economic activities, and improving the happiness of migrant workers has become a necessary measure for China to promote ongoing new-type urbanization. However, the “urbanization” of migrant workers is not only a cost problem but also a social integration problem that includes psychological, behavioral, and identity factors [5]. The social services accompanying urban household registration, such as education, medical care, and advantages in the labor market, all mean that “incomplete urbanization” will decrease the happiness of migrant workers compared to that of local urban residents. However, in recent years, the Chinese government has combined policies such as homesteads and contracted land with rural household registration. Farmers with a rural household registration can also enjoy benefits like the village collective dividend. As a result, a considerable number of migrant workers are willing to retain their rural household registration to work and live in cities and enjoy the benefits accompanying rural household registration. This phenomenon has created obstacles to new-type urbanization in China. It has also raised questions about whether the conversion of household registration has actually improved the happiness of migrant workers.

The question of whether the conversion of household registration has improved the happiness of migrant workers has a vital role in improving the overall happiness of Chinese citizens and embodying the “people-oriented” core of the new urbanization. The present study uses this question to explore the internal connections and interactions related to the happiness of migrant workers, the conversion of household registration, and the characteristics of migrant worker groups. Based on these results, suggestions are made to improve the happiness of migrant workers and promote new-type urbanization in China more effectively.

## 2. Theoretical Framework

There are many global studies on urbanization and household registration reform. These studies reflect the relationship between household registration status and the happiness of migrant workers from an outside perspective. However, due to the complexity of China’s household registration system and the particular social environment in which migrant workers live, there is no uniform conclusion as to which type of household registration makes migrant workers happiest.

The negative impact of rural household registration on the happiness of migrant workers is mainly reflected in the fact that China’s household registration system is not just a piece of “paper” but instead represents the inequality of access to various rights, such as social security, between urban and rural residents [6]. Due to China’s unique urban–rural dual management structure, the Chinese government attaches importance to the development of cities but neglects the development of rural areas. The main problems encountered by migrant workers after entering a city to work are the various policies and regulations from local governments, which severely reduce the sense of belonging among migrant workers and have made the migrant workers force institutionally excluded in cities [7]. Because of this exclusion, there is a clear gap between migrant workers and urban residents in their social and political activities [8]. This gap is reflected in the income of the residents, which can directly explain the 28% urban–rural income gap. This income gap further leads to differences in the happiness between migrant workers and local residents [9]. At the same time, China’s unique household registration system has certainly affected the happiness of migrant workers in terms of their social support, social trust, and freedom [10]. The mechanism of the conversion of household registration to improve the happiness of migrant workers is mainly carried out through the acquisition of “urban security”. This is done by increasing the level of an individual’s resistance to risks [11], thereby creating a gap in the happiness of migrant workers with different household registration statuses.

However, urban household registration also has a negative impact. Most of the rights associated with a rural household registration will be lost with a conversion to urban household registration, especially rights such as contracted land and collective dividends. These fundamental rights provide economic security for migrant workers to return to rural areas in the future. At the same time, the conversion of household registrations does not mean that migrant workers can achieve stable employment in the city. Due to the excessively high price levels in large cities and the fierce competition in the labor market, a worker’s ability to absorb low-end labor has been reduced. The living and working pressures of migrant workers in the city and the inconsistency between supply and demand in the low-end labor market in cities have made urban household registration less attractive to migrant workers [12]. Further, the gap between public services and social security in small and medium-sized towns and rural areas is low. Although these aspects of large cities are far ahead, their costs are too high. A national survey in China showed that less than 20% of farmers are willing to settle in small and medium-sized towns near their household registration area [13]. The essential reason for the decline in the attractiveness of urban household registration is the reverse change in the relative value of urban and rural household registration. At present, in terms of the distribution of benefits in China, rural household registration has more advantages. This phenomenon is mainly due to China’s policy shift toward “agriculture, rural areas, and farmers” in recent years. These factors have led to the weakening of the traditional advantages of urban household registration and have made urban household registration less attractive to migrant workers [14].

**Figure 1:**
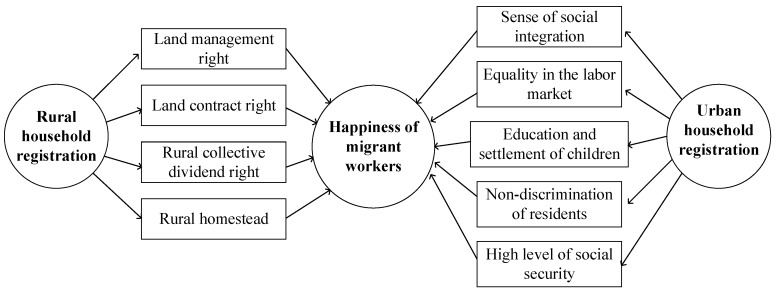
The Influence Paths.

Combining the above research on the effects of household registration conversion on the happiness of migrant workers, we can extract the influence paths for different types of household registration on the happiness of migrant workers, as shown in Figure 1.

As can be seen from Figure 1, urban household registration has improved happiness through aspects of urban integration, advantages of the labor market, resources for urban education, non-discrimination by local residents, and high levels of social security. Additionally, rural household registration can improve happiness through land contracting right, land management right, collective dividend right, and rural homestead.

This study found that previous research suffered from several problems when exploring the relationship between household registration status and happiness. First, most of these studies used a single question (“Are you happy?”) as the sole criterion for judging happiness [15]. It is, however, difficult to reflect the happiness state of migrant workers through a single question. In particular, migrant workers are a group with a relatively complicated living environment and group structure. This is not a situation that can be summed up with one question. Therefore, the stability of the results of these studies is not sufficient to explain the various effects of the conversion of household registration on the happiness of migrant workers. Second, due to the insufficient sample size of the migrant workers who converted their household registration in some studies, the results of these studies are less stable and not generalizable [16]. Third, the research objects of these studies are all farmers with rural household registrations. They also did not distinguish between farmers and migrant workers because the farmer group is too large and because many farmers working and living in rural areas are not affected by household registration status. Migrant workers comprise only a portion of farmers. These factors make the results of these studies insufficient for a comprehensive and practical judgment of the relationship between household registration status and the happiness of migrant workers.

In order to focus on the interconnection between the conversion of household registration and the happiness of migrant workers, the structure of this paper is as follows. First, we use the data from the China Migrants Dynamic Survey (CMDS) to select a sample of migrant workers living and working in cities. Through theoretical research and a summary of previous studies, we select multiple indexes that can reflect the happiness of migrant workers based on the data of CMDS. Then, we use principal component analysis (PCA) to reduce the dimensions of the indexes and obtain a comprehensive index that reflects the happiness of migrant workers; this index avoids the one-sidedness and instability of the research results caused by using only one question to measure happiness. Later, using this happiness index as the dependent variable, propensity score matching (PSM) was used to explore the effect of the conversion of household registration on the happiness of migrant workers. In this way, the self-selection of samples can be fully considered to more accurately answer the question of whether the conversion of household registration improves the happiness of migrant workers. Finally, the study uses group difference analysis to group migrant workers with different characteristics in order to investigate the impact of the conversion of household registration on the happiness of migrant workers with different characteristics and explore the resulting biases caused by the differences in the characteristics of the migrant worker samples.

## 3. Model Description and Data Analysis

### 3.1. Construction of the Propensity Score Matching (PSM) Model

Because the conversion of household registration is not entirely random in China, it can be “self-selected” by the sample to a certain extent. This situation may cause severe endogenous problems. Moreover, the data span one year and are cross-sectional. It is thus impossible to compare the happiness of the same migrant worker with different household registration statuses. Therefore, this study applies the propensity score matching (PSM) model and selects the migrant workers whose covariate characteristics are as close as possible but have different household registration statuses to create a “counterfactual” effect. In this way, we can eliminate the errors caused by endogenous problems to more accurately estimate the impact of the conversion of household registration on the happiness of migrant workers.

The principle of PSM is matching estimation. In the control group with rural household registration, we find sample A and make that sample as similar as possible to the personal characteristics of sample B in the treatment group whose household registration has changed from rural to urban, so A≈B. In this way, the effect of the conversion of household registration on the happiness of migrant workers can be approximated by the happiness of sample B minus the happiness of sample A. In this way, we established the following model to estimate the impact of the conversion of household registration on the happiness of migrant workers:

(1) $$F_{i}^{d}=\alpha X_{i}+\beta_{i}D_{i}+\varepsilon_{i}$$

where $\;F_{i}^{d}$ represents the happiness of the i-th migrant worker, coefficient α represents the influence coefficient of individual characteristics, covariate $X_{i}$ represents the observable individual characteristic of the i-th migrant worker, $D_{i}$ indicates the household registration status of the i-th migrant worker, $D_{i}=1$ indicates that the migrant worker’s household registration has changed from rural to urban, $D_{i}=0$ indicates that the migrant worker’s household registration is rural, coefficient $\beta_{i}$ indicates the degree of influence of the household registration identity conversion on the happiness of the migrant workers, and $\varepsilon_{i}$ is a random distribution term.

### 3.2. Data Source

The data in this study are derived from the CMDS released by the China National Health Commission in 2017, which is a single cross-section dataset. This survey covered all 31 provinces of China, and the samples were chosen randomly. The probability proportional to size (PPS) sampling technique was used for this survey. To study the effect of the conversion of household registration on the happiness of migrant workers, a sample of migrant workers aged 16–60 years was selected from the samples surveyed by the urban resident committee. There are two types of samples: One includes rural migrant workers with rural household registration, and the other includes rural migrant workers who have carried out the conversion of their household registration. Surveying according to the urban resident committee ensured that these migrant workers work and live in cities. The age of 16 to 60 years conforms to the definition of labor age in Chinese laws. A total of 81,381 samples were included in this study, among which 5147 were migrant workers whose household registrations changed from rural to urban, and 76,234 were migrant workers whose household registrations were rural.

### 3.3. Variable Settings and Descriptive Statistics

The selection of variables in the PSM model is particularly important and primarily includes the selection of dependent variables, independent variables, and covariates. The independent variable here is household registration status. The sample selected in this study is of two types. One is the migrant worker with rural household registration, and the other is the migrant worker whose household registration has changed from rural to urban. The dependent variable is the happiness of the migrant workers. The covariates are the personal characteristics of the migrant workers related to the conversion of household registration and the workers’ happiness. Moreover, the covariates need to satisfy the conditional independent assumptions, common support conditions, and the balancing hypothesis at the same time.

In the selection of covariates, based on the relevant literature, we determined the basic characteristics of individuals, such as age, education, and health; the characteristics of work, such as absolute income, relative income, unemployment, and nature of work; and social characteristics, such as social trust, social capital, social participation, social ownership, and government expenditure. These characteristics have an important impact on residents’ happiness [17,18]. Based on the CMDS and the particularity of the research object in this study, there are three types of characteristics that can affect the objective characteristics of the happiness of migrant workers. The basic characteristics of the individual include variables such as age, gender, educational background, marital status, and health status. The characteristics of work include current employment status, total monthly expenditures, and total income of the family, whether there is a contracted land in their hometown, whether they have their collective dividends, their hours worked this week, and their monthly income changes compared to the same period last year. Finally, we selected social characteristics that reflect the basic social situation of the city, including whether they establish the resident health files, whether the residents participate in the new rural cooperative medical insurance, whether they apply for personal social security cards, and whether they apply for a temporary residence permit. The variable settings and descriptive statistics are shown in Table 1 below. Since the selection of the above variables mainly considers the relationship with happiness, this study uses OLS and Logit models to study the relationship between these variables and the conversion of household registration again to verify the rationality of the variable selection and ensure the stability of the PSM results. Table 1 presents descriptions and definitions of the variables.

## 4. Measuring the Happiness of Migrant Workers

### 4.1. Selecting the Evaluation Index for the Happiness of Migrant Workers

This study refers to the research on happiness by other researchers. This research determined that the residents’ self-evaluation of their subjective welfare, economic status, their economic status between different years, expectations for their future economic situation, daily emotional state, social justice perception, social security cognition, and other factors jointly determine their happiness [19,20,21,22]. At the same time, due to the particularity of the migrant worker group, senses of social integration and social fairness are closely related to happiness [23,24]. Since happiness is subjective, this study, based on the above summary and considering the particularities of the migrant workers in the city, uses the migrant workers’ self-evaluation of their work, family, and social conditions from the CMDS in 2017, yielding a total of 12 indicators to comprehensively reflect their subjective happiness (Table 2).

According to the descriptive statistics, we can see that problems such as low income and taking care of the elderly remain important reasons for the lack of happiness among migrant workers. In other respects, migrant workers are satisfied with their lives.

### 4.2. Measurement of the Happiness of Migrant Workers

Based on the results in Table 3, we combined the above indicators and used the Stata 14 (StataCorp LLC, College Station, TX, USA) to construct a PCA model. Firstly, we performed a Kaiser–Meyer–Olkin (KMO) test and a Bartlett spherical test to verify whether the selected indicators are suitable for the PCA. It can be seen that the result of the Kaiser–Meyer–Olkin Measure of Aampling adequacy is good, and the significance value of Bartlett’s Test of Sphericity is *p* < 0.001, which indicates that the correlation between the indicators is high and that the independence is weak. The results show that the above indicators are suitable for PCA. We then applied PCA to construct a cumulative variance table.

Based on the analysis results, the sum of the variance contribution ratios of the first five principal components reached 70.292%, indicating that the five indicators can cover most of the original 12 indicators and can be used to evaluate the happiness of migrant workers. We then use Formula (2) to calculate the comprehensive happiness index f of migrant workers in the city:

(2) $$\begin{array}{l} f=\left(22.630\%/70.292\%\right)F_{1}+\left(18.023\%/70.292\%\right)F_{2}\\ \;\;+(11.979\%/70.292\%)F_{3}+\left(9.851\%/70.292\%\right)F_{4}\\ \;+\left(7.809\%/70.292\%\right)F_{5.} \end{array}$$

Since f has a negative value, formula (3) is used:

(3) F=f+|min(f)|.

The final happiness degree, F, of the migrant workers is thus calculated, and the descriptive statistics of $F_{1},\;F_{2},\;\;F_{3},\;\;F_{4},\;F_{5},\;\;f$, and F are shown in Table 4. F indicates the happiness of the migrant workers. The higher the value of F, the higher the happiness of the migrant workers in the city. The results show that the average happiness index of the migrant workers who changed their household registration status is 3.708, while the average happiness index of the migrant workers who did not change their household registration status is 3.603. Thus, there may be a positive correlation between the conversion of household registration and the happiness of migrant workers.

## 5. Results

### 5.1. Testing Results of the Correlation of Variables

OLS models are usually used to calculate the impact degree, so we first check whether the OLS model is suitable for this study. We use the OLS model to calculate the effect of the conversion of household registration on the happiness of the migrant workers (Model 1) and then add covariates to test the significance of the covariates (Model 2). The results are shown in Table 5.

The results of Model 1 show that the effect of the conversion of household registration on the happiness of migrant workers is positive and significant. However, the value of $R^{2}$ is 0.001, indicating that the degree of the fit is insufficient. After adding covariates, the results of Model 2 show that the effect of the conversion of household registration on the happiness of migrant workers is still positive and significant. Except for gender and health status, the remaining covariates all passed the 10% significance test, which indicates that this study has high rationality in the selection of its covariates. The value of $R^{2}$ in Model 2 is 0.031. Although this value is improved compared to the model’s, the model’s fit is still insufficient. Therefore, this study adopts the PSM model.

### 5.2. Logit Estimates Before PSM

Before using the PSM model, it is necessary to use the Logit model to estimate the impact of covariates on the conversion of the household registration of migrant workers. Because the Logit model is used to match propensity scores, the rationality of its setting is particularly important. The covariates are divided into three categories: basic personal characteristics, characteristics of work, and social characteristics. In this way, a total of four logit models are established to analyze the influencing factors of rural household registration conversion (Table 6). Model 1 primarily controls the variables that reflect the basic personal characteristics for migrant workers, excluding variables that are not significant in the OLS model. Model 2 features variables for characteristics of work. Model 3 adds the variables for social characteristics based on the previous model. Based on Model 3, we add the previous insignificant variables to obtain Model 4.

The goal of setting up four logit regression models is to determine an optimal model to calculate the value of PS for sample matching. Because there is no clear way to evaluate the Logit model, this paper uses $\mathrm{Pseudo}\;R^{2}$, AUC, and CLAS to assess and compare the model. According to the results, in addition to the CLAS indicator, the other two indicators show that model four is the most suitable. Therefore, when using the PSM model, this study chooses Model 4 to estimate the value of PS. In Model 4, all the variables passed a 5% significance test. According to the results and the assignment of variables, it can be seen that women are more inclined to carry out a conversion of household registration. An increase in age, an improvement of educational background, good health status, an increase in income and expenditures, an increase in working hours, an increase in income over the same period, the establishment of resident health records, and the handling of a temporary residence permit have positive effects on promoting the conversion of household registration. The factors that restrict the conversion of household registration include contracted land, collective dividends, participation in the new rural cooperative medical insurance, and the handling of individual social security cards.

### 5.3. Application of the PSM Model

In order to ensure the high quality of the matching results and the reliability of the estimated results, it is necessary to verify the common support conditions and the balancing hypothesis. The common support condition ensures that the samples in the treatment group can be matched with the samples in the control group through their propensity scores. The balancing hypothesis ensures that, in addition to the differences in the household registration of migrant workers, there is no significant difference in the other characteristics of the migrant workers in the treatment group and the control group. That is, the balancing hypothesis ensures that the happiness of the migrant workers is only affected by household registration.

#### 5.3.1. The Common Support Conditions

**Figure 2:**
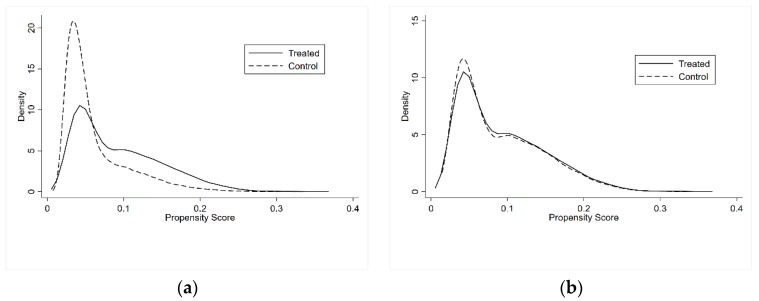
Before (**a**) and After (**b**) One-to-one Nearest Neighbor Matching.

Figure 2 shows the kernel density distributions of the treatment group and the control group before and after one-to-one nearest neighbor matching of the samples. There is a significant difference in the propensity scores of the two groups of samples before matching. After matching, the kernel density curves between the two groups almost overlap, indicating that there is no significant difference in the characteristics of the migrant workers in the two groups of samples after matching.

#### 5.3.2. The Balancing Hypothesis

Table 7 presents the results of the balance test. The results of the T-test are not significant after matching, which indicates that the difference between the treatment group and the control group is not obvious after matching. Thus, the balancing hypothesis is verified.

#### 5.3.3. Results of the PSM

Table 8 presents the results of the propensity score matching. Four methods (one-to-one nearest neighbor matching, 4-nearest neighbor matching, nearest-neighbor matching with a caliper, and kernel matching) were used to estimate the effect of impact.

The results show that after one-to-one nearest neighbor matching, the value of the average treatment effect on the treated (ATT) is 0.0589. This result passes the 5% significance test, indicating that the happiness of the migrant workers can be improved when their household registration is changed from rural to urban. The ATT values obtained by the other three matching methods are almost the same, which shows that the analysis results do not change with a change in the matching methods and remain relatively stable.

### 5.4. Analysis of Group Difference

Although the results of the PSM model are significant, the value of the ATT is small, indicating that the conversion of household registration offers a limited improvement to the overall happiness of migrant workers. Moreover, because ATT can only represent the average improvement in the happiness of migrant workers after the conversion of household registrationit cannot show the structural differences between migrant workers groups. Therefore, exploring group differences among migrant workers can help explain why the conversion of household registration has a small effect on the overall happiness of migrant workers. At the same time, focusing on the ATT value of migrant workers with different characteristics could make the research results more targeted and comprehensive.

This study selected five characteristics (age, educational background, whether there is contracted land in their hometown, whether they have their collective dividends, and the total monthly income of the family) to divide the migrant worker groups. These characteristics are not only related to migrant workers’ willingness to change their household registration but also profoundly affect their happiness. Among them, age and educational background, essential variables of human capital, are included in many cities’ criteria to measure whether a worker is eligible for the conversion of his or her household registration, which is the objective condition for migrant workers to carry out the conversion of their household registration. At the same time, due to their different ages and educational backgrounds, migrant workers have different perceptions of happiness, which will affect their happiness.

The three variables of whether there is contracted land in their hometown, whether they have their collective dividends, and the total monthly family income are the subjective motivation factors that determine whether the migrant workers will carry out the conversion of their household registration. Migrant workers who have contracted land in their hometown may not want to give up the land benefits accompanying their rural household registration, thereby reducing their desire to convert their household registration. By the same token, migrant workers with their collective dividends will lose this part of their income once their household registration is converted, which is also an important factor limiting the conversion of household registration. Total monthly household income is regarded as the most critical factor for migrant workers to choose to work in cities. Moreover, an increase in income will not only make migrant workers want to stay in cities to change their household registration status but will also improve their happiness. Therefore, it is necessary to group migrant workers according to their age, educational background, whether they have contracted land in their hometown, whether they have their collective dividends, and the total monthly income of their families. Focusing on the ATT value and the average happiness of different groups of migrant workers will provide a theoretical basis for accelerating the process of new-type urbanization and improving worker happiness. This study uses one-to-one nearest neighbor matching to determine the effect of converting household registration on the happiness of different groups of migrant workers. The results are shown in Table 9.

Based on the previous results of the Logit model, we can know that an increase in age can prompt migrant workers to convert their household registration. The group difference analysis reflects that the average happiness of migrant workers increases with age and reaches its highest level after 45 years of age. The value of ATT increases first and then decreases with age, reaching its maximum from age 35 to 45 years. The reason for this phenomenon is that, after the age of 45, most migrant workers’ children will have reached adulthood. The improvement of their work skills, the intensity of their work, and the gradual improvement of their working environments produce the highest average happiness. The 35–45-year-old migrant workers are at a stage where they take care of both the elderly and their children. Due to the discrimination of rural household registration in the labor market and social integration issues, the conversion of household registration has the most significant effect on the promotion of happiness at this age.

Educational background is also an essential factor for promoting the conversion of household registration. The analysis of group differences reflects that the average happiness of migrant workers generally increases with an improvement of their educational background. This result is mainly due to the positive correlation between educational background and individual abilities. Educational background is an essential part of human capital. By improving migrant workers’ educational backgrounds, their working environment and social status also improve. These factors have led to a positive correlation between educational background and happiness. However, the ATT value decreases with an improvement in educational background. This situation occurs because the migrant workers with a lower educational background experience a worse social environment and working environment and suffer greater discrimination against their rural household registration. Migrant workers with a high educational background can reduce the negative impact of their rural household registration through their abilities and educational backgrounds. This phenomenon runs counter to the household registration policies of many cities in China, and many cities take educational background as an important criterion for the conversion of household registration. However, this study finds that, as the educational background of migrant workers increases, the effect of the conversion of household registration on the happiness of migrant workers continues to decline; likewise, the attractiveness of urban household registration also gradually decreases.

The average happiness of migrant workers with contracted land in their hometown is higher, and the improvement of their happiness by converting their household registration is significantly more positive than that of migrant workers without contracted land in their hometown. The main reason for this phenomenon is that Article 26 of the China Land Contracting Law stipulates that during the contracting period, local government shall not recover the contracted land. China’s rural land contract system is based on households. If farmers move to cities, the original reserved land in the countryside is retained, and their families continue to cultivate and contract that land. The implementation of this policy increases the effect of converting household registration on migrant workers with contracted land. These workers can not only retain the rights to their contracted land but can also enjoy the public social services offered by their urban household registration. Therefore, the average happiness and ATT value of migrant workers with contracted land are higher than those of workers without contracted land.

The average happiness of migrant workers with collective dividends is high, but the ATT value is low, which is contrary to the situation of contracted land. This phenomenon occurs because the Chinese government stipulates that collective dividends in villages can only be given to farmers with rural household registrations. Once a farmer’s household registration is converted, this right will be forfeited. Therefore, migrant workers with collective dividends have a lower willingness to convert their household registration, as their happiness will decrease after the conversion. Therefore, China’s household registration reform should consider the phenomenon of collective dividends.

The average happiness of migrant workers increases significantly with an increase in total monthly family income. High income has an observably positive effect on happiness. In the Logit model, with an increase in income, migrant workers are more willing to convert their household registration. However, the effect of converting household registration on happiness decreases with an increase in income. The main reason for this phenomenon is similar to the situation of educational background. As the income of migrant workers in the city reaches a higher level, the restrictions and adverse effects of rural household registration will become increasingly fewer. Therefore, the impact on happiness for high-income migrant workers is limited, as the desire to convert household registration is mainly reserved for low-income migrant workers.

## 6. Conclusions

This study selects self-evaluation indicators that are closely related to the lives of migrant workers to measure their happiness. Our research on the effects of the conversion of household registration on the happiness of migrant workers found that the conversion of household registration actually improved happiness, but this improvement was limited. According to the results of the Logit model, women are more inclined to carry out the conversion of household registration. Age growth, improvement in educational background, health status, increases in income and expenditures, increases in working hours, increases in income over the same period, the establishment of resident health records, and the handling of temporary residence permits have positive effects on promoting the conversion of household registration. The group difference analysis found that the group of migrant workers, aged 45 years and above, who have a bachelor’s degree, contracted land in their hometown, collective dividends, and a monthly family income of more than 8000 yuan have the highest average happiness. The group of migrant workers, aged 30–45 years, who did not attend primary school, have contracted land in their hometown, have no collective dividends, and have a monthly family income of 4000–6000 yuan showed the highest value of ATT.

The results of this analysis show that the conversion of household registration has, indeed, improved the happiness of migrant workers. Different groups of migrant workers are affected to varying degrees, and the conversion of household registration even has a negative effect on some migrant workers. This result shows that household registration status, the happiness of migrant workers, and the characteristics of migrant worker groups are closely linked. On the one hand, this result means that the “household registration gap” precipitated by China’s household registration system is still apparent. The welfare accompanying resident household registration always makes the happiness of migrant workers with rural household registration low. On the other hand, this result also provides a strong basis for China to promote the reform of its household registration system firmly and, at the same time, explains some problems in the implementation of the relevant policies. This result means that China’s household registration system reform needs to implement different household registration policies for different groups of migrant workers to improve the happiness of each group and to continue to promote the construction of new-type urbanization. Thus, we propose the following policy recommendations.

(1) Since the conversion of household registration has a positive effect on the happiness of migrant workers, China should continue to deepen its reform of the household registration system. On the one hand, the Chinese government should simplify the difficulty of naturalization and gradually reduce the settlement standards for big cities. At the same time, the settlement restrictions on small and medium-sized cities should be fully relaxed. These government measures could also provide cities with sufficient labor. On the other hand, the urban public services and social security accompanying resident household registration should be gradually reduced so that migrant workers and residents can achieve equal and fair lives in the city. Only in this way can migrant workers genuinely integrate into urban life and achieve the “people-oriented” aims of modern construction.

(2) According to the analysis results, with an increase in the family’s monthly income, the happiness of migrant workers significantly improves, and an increase in income dramatically promotes the conversion of household registration. Therefore, in the process of promoting new-type urbanization, fairer treatment should be given to migrant workers in the labor market. Enterprises and public institutions should treat migrant workers with rural household registrations equally when recruiting them, and migrant workers should enjoy the same treatment as urban residents in terms of their income. Income is the main goal for migrant workers who choose to work in cities. Only by steadily increasing the income of these works can we effectively improve their happiness.

(3) The happiness of migrant workers increases significantly with an improvement in educational background. Moreover, the value of the ATT of migrant workers with a low educational background is higher. Therefore, on the one hand, we should strengthen our investment in resources for rural education, continue to popularize nine-year compulsory education and high school education, and in urban areas, protect the rights for migrant workers’ children to obtain an education. Local governments should enable migrant workers and their children to enjoy the educational resources of cities fairly and improve their human capital. On the other hand, local governments should reduce restrictions on the educational backgrounds of migrant workers when implementing household registration policies in big cities. Indeed, the happiness of high-education migrant workers is hardly affected by the conversion of their household registration. Therefore, in the process of new-type urbanization, attention should be paid to low-education migrant workers. The difficulty in converting one’s household registration should be reduced to improve the average happiness of all migrant workers.

(4) Because contracted land and collective dividends have different policies for the conversion of household registration, in this study, they exert opposite effects on the happiness of migrant workers. In the process of household registration reform in China, we should pay attention to the retention of the benefits associated with rural household registration in order to increase the willingness of migrant workers to convert their household registration and to enable the conversion of household registration to have a positive effect on their happiness.

(5) Finally, we should respect the will of the residents. For rural migrants workers who cannot or do not want to settle in the city, we should improve the temporary residence permit system and ensure that urban public services and social security are no longer only related to household registration.

Our study has some limitations. Due to the nature of the data obtained, this study only conducted cross-sectional data analysis. Panel data analysis may lead to different results. Further, the selection of various indicators in this study mainly depended on previous research results.

## Figures and Tables

**Table 1:** Descriptions and definitions of the variables.

Variable Type	Variable Name	Variable Assignment	Mean	Std
Independent variable	Household registration status	0 = Rural; 1 = Rural to urban	0.063	0.243
Dependent variable	Happiness of migrant worker	Continuous variable	3.609	0.707
Basic personal characteristics	Age	1 = 16–30 years old; 2 = 30–45 years old; 3 = 45 years and older	1.813	0.707
	Gender	1 = Male; 2 = Female	1.439	0.496
	Educational background	1 = No school; 2 = Primary school; 3 = Junior middle school; 4 = Senior middle school/technical secondary school; 5 = College; 6 = University; 7 = Graduate school	3.409	1.059
	Marital status	1 = Unmarried; 2 = First marriage; 3 = Remarriage; 4 = Divorced; 5 = Widowed; 6 = Cohabitation	1.921	0.615
	Health status	1 = Health; 2 = Basic health; 3 = Ill health, but they can take care of themselves; 4 = Can’t take care of themselves	1.171	0.412
Working characteristics	Current employment status	1 = Employees with regular employers; 2 = Employees without regular employers; 3 = Employer; 4 = Self-management; 5 = Other	2.419	1.426
	The total monthly expenditure of the family	1 = Under 2000 yuan; 2 = 2000–4000 yuan; 3 = 4000–6000 yuan; 4 = More than 6000 yuan	2.090	0.924
	The total monthly income of the family	1 = Under 4000 yuan; 2 = 4000–6000 yuan; 3 = 6000–8000 yuan; 4 = More than 8000 yuan	2.407	1.122
	Whether there is contracted land in their hometown	1 = No; 2 = Unclear; 3 = Yes	2.141	0.954
	Whether they have their collective dividends distributed	1 = No; 2 = Unclear; 3 = Yes	1.114	0.389
	Hours worked this week	1 = Under 40 h; 2 = 40–60 h; 3 = 60–80 h; 4 = More than 80 h	2.311	0.970
	Monthly income changes compared to the same period last year	1 = Reduce; 2 = Not suitable; 3 = Basically unchanged; 4 = Increase	2.419	1.103
Social characteristics	Whether they establish the resident health files	1 = No, I haven’t heard of it; 2 = Unclear; 3 = No, but I’ve heard of it; 4 = Yes	2.494	1.168
	Whether they participate in the new rural cooperative medical insurance	1 = No; 2 = Unclear; 3 = Yes	2.429	0.890
	Whether they apply for personal social security cards	1 = No, I haven’t heard of it; 2 = Unclear; 3 = No, but I’ve heard of it; 4 = Yes	3.136	1.053
	Whether they apply for the temporary residence permit	1 = No; 2 = Unclear; 3 = Yes	2.328	0.941

**Table 2:** Descriptive Statistics for the Happiness Index of Migrant Workers.

Index Type	Index Content	Assignment Status	Mean	Std
Working conditions	Do you think your family income is too low?	1 = Yes; 2 = Unclear; 3 = No	1.72	0.71
	Do you think it is difficult for you to find a stable job?	1 = Yes; 2 = Unclear; 3 = No	2.15	0.75
Family conditions	Do you think there are problems with your children’s education?	1 = Yes; 2 = Unclear; 3 = No	2.20	0.73
	Do you think it is difficult to look after your children?	1 = Yes; 2 = Unclear; 3 = No	2.24	0.60
	Do you think it is difficult for you to bear the educational expenses of your children?	1 = Yes; 2 = Unclear; 3 = No	2.27	0.59
	Do you think it is difficult for you to support the elderly?	1 = Yes; 2 = Unclear; 3 = No	1.81	0.62
Social conditions	Is your home looked down upon by the local people?	1 = Yes; 2 = Unclear; 3 = No	2.45	0.61
	Does your family not adapt to the local living habits?	1 = Yes; 2 = Unclear; 3 = No	2.48	0.59
	Are you willing to join the local people and become one of them?	1 = Totally no; 2 = No; 3 = Basic yes; 4 = Totally yes	3.34	0.63
	Do you think the locals are willing to accept you as one of them?	1 = Totally no; 2 = No; 3 = Basic yes; 4 = Totally yes	3.28	0.61
	Do you like the city where you live now?	1 = Totally no; 2 = No; 3 = Basic yes; 4 = Totally yes	3.40	0.57
	Do you care about the city where you live now?	1 = Totally no; 2 = No; 3 = Basic yes; 4 = Totally yes	3.36	0.60

**Table 3:** Cumulative Variance Table.

Component	Initial Eigenvalue	Percentage of Variance	Grand Total (%)
F1	2.716	22.630	22.630
F2	2.163	18.023	40.653
F3	1.437	11.979	52.632
F4	1.182	9.851	62.483
F5	0.937	7.809	70.292
Kaiser–Meyer–Olkin Measure of Sampling Adequacy		0.70	
Bartlett spherical test	Approx. Chi-Square	233599.87	
	df	66.00	
	Significance	0.00	

**Table 4:** Principal Component Descriptive Statistics.

Principal Component	Mean	Std	Min	Max
F1	2.29 × 10^−8^	1.647	−8.275	2.578
F2	9.81 × 10^−10^	1.471	−5.275	4.094
F3	1.12 × 10^−8^	1.198	−4.601	4.189
F4	−3.11 × 10^−8^	1.088	−3.608	4.237
F5	1.19 × 10^−8^	0.967	−3.492	3.526
f	4.56 × 10^−9^	0.707	−3.609	1.838
F	3.609	0.707	0.000	5.447

**Table 5:** Results of the OLS model with the Migrant Workers’ Happiness as the Dependent Variable.

Variable Name	Model 1		Model 2	
	Coef.	*p*	Coef.	*p*
Household registration status	0.105	0.000	0.067	0.000
Age			0.056	0.000
Gender			−0.003	0.586
Educational background			0.045	0.000
Marital status			0.008	0.059
Health status			−0.008	0.181
Current employment status			0.029	0.000
The total monthly expenditures of the family			0.034	0.000
The total monthly income of the family			−0.006	0.052
Whether there is contracted land in their hometown			0.041	0.000
Whether they have their collective dividends			−0.026	0.000
Hours worked this week			0.016	0.000
Monthly income changes compared with the same period last year			−0.021	0.000
Whether they establish the resident health files			0.048	0.000
Whether they participate in the new rural cooperative medical insurance			−0.038	0.000
Whether they apply for personal social security cards			0.021	0.000
Whether they apply for a temporary residence permit			0.005	0.087
_cons	3.603	0.000	3.069	0.000
Number of obs	81381.000		81381.000	
R-squared	0.001		0.031	

**Table 6:** Logit Model with Household Registration as the Dependent Variable.

Variable Name	Model 1	Model 2	Model 3	Model 4
Age	0.1173 ***	0.1549 ***	0.1087 ***	0.1307 ***
	(0.0231)	(0.0235)	(0.024)	(0.0244)
Gender				0.0712 **
				(0.03)
Educational background	0.3730 ***	0.3307 ***	0.2485 ***	0.2492 ***
	(0.0137)	(0.0149)	(0.0153)	(0.0154)
Marital status	0.1246 ***	0.0976 ***	0.0814 ***	0.0824 ***
	(0.0227)	(0.0237)	(0.0242)	(0.0242)
Health status				−0.1923 ***
				(0.041)
Current employment status		−0.0921 ***	−0.0451 ***	−0.0458 ***
		(0.012)	(0.0123)	(0.0123)
The total monthly expenditures of the family		0.0668 ***	0.0404 **	0.0448 **
		(0.0199)	(0.0202)	(0.0202)
The total monthly income of the family		0.1060 ***	0.0913 ***	0.0862 ***
		(0.0168)	(0.0172)	(0.0172)
Whether there is contracted land in the hometown		−0.1347 ***	−0.0991 ***	−0.0937 ***
		(0.0152)	(0.0154)	(0.0155)
Whether they have their collective dividends		−0.1072 ***	−0.1092 ***	−0.1092 ***
		(0.039)	(0.0394)	(0.0395)
Hours worked this week		0.1317 ***	0.1842 ***	0.1885 ***
		(0.0168)	(0.0171)	(0.0172)
Monthly income changes compared to the same period last year		0.1041 ***	0.0920 ***	0.0898 ***
		(0.0147)	(0.0149)	(0.0149)
Whether they establish the resident health files			0.1223 ***	0.1194 ***
			(0.0127)	(0.0128)
Whether they participate in the new rural cooperative medical insurance			−0.5044 ***	−0.5043 ***
			(0.0161)	(0.0161)
Whether they apply for personal social security cards			−0.0443 ***	−0.0443 ***
			(0.0158)	(0.0158)
Whether they apply for a temporary residence permit			0.1013 ***	0.1012 ***
			(0.0165)	(0.0165)
Pseudo R2	0.0193	0.0285	0.0584	0.0591
AUC	0.6023	0.6283	0.6889	0.6902
N	81,381	81,381	81,381	81,381
CLAS	93.68%	93.68%	93.68%	93.68%

Note: ** *p* < 0.05, *** *p* < 0.01, the standard deviation shown in parentheses is robust standard error. AUC is the area under the curve of ROC; CLAS stands for Classification and is used to indicate the accuracy of the logit model’s predictions.

**Table 7:** The Results of the Balance Test.

Variable Name	Unmatched/Matched	Mean		t-Test	
		Treated	Control	t	*p*
Age	Unmatched	1.7789	1.8156	−3.60	0.000
	Matched	1.7787	1.7837	−0.39	0.700
Gender	Unmatched	1.4552	1.4376	2.46	0.014
	Matched	1.4553	1.4543	0.10	0.921
Marital status	Unmatched	1.9421	1.9198	2.52	0.012
	Matched	1.9419	1.9366	0.45	0.650
Educational background	Unmatched	3.7894	3.3834	26.73	0.000
	Matched	3.7890	3.7674	0.95	0.343
Marital status	Unmatched	1.1313	1.1736	−7.13	0.000
	Matched	1.1314	1.1205	1.55	0.121
Current employment status	Unmatched	2.1888	2.4343	−11.96	0.000
	Matched	2.1891	2.1949	−0.21	0.834
The total monthly expenditures of the family	Unmatched	2.2553	2.0791	13.25	0.000
	Matched	2.2553	2.2301	1.36	0.173
The total monthly income of the family	Unmatched	2.6408	2.3916	15.44	0.000
	Matched	2.6407	2.6304	0.47	0.637
Whether there is contracted land in their hometown	Unmatched	2.0235	2.1490	−9.14	0.000
	Matched	2.0237	2.0282	−0.23	0.815
Whether they have their collective dividends	Unmatched	1.1057	1.1145	−1.56	0.118
	Matched	1.1057	1.0921	1.90	0.058
Hours worked this week	Unmatched	2.2662	2.3144	−3.46	0.001
	Matched	2.2658	2.2925	−1.40	0.161
Monthly income changes compared to the same period last year	Unmatched	2.6108	2.4056	12.93	0.000
	Matched	2.6106	2.6275	−0.81	0.420
Whether they establish the resident health files	Unmatched	2.6878	2.4814	12.28	0.000
	Matched	2.6875	2.7188	−1.34	0.180
Whether they participate in the new rural cooperative medical insurance	Unmatched	1.9376	2.4627	−41.37	0.000
	Matched	1.9378	1.9518	−0.72	0.473
Whether they apply for personal social security cards	Unmatched	3.2978	3.1249	11.41	0.000
	Matched	3.2977	3.3247	−1.40	0.161
Whether they apply for a temporary residence permit	Unmatched	2.4350	2.3211	8.41	0.000
	Matched	2.4353	2.4582	−1.30	0.192

**Table 8:** The Results of the PSM.

Dependent Variable	Matching Method	ATT	S.E.	T-Stat
Happiness of migrant workers	One-to-one nearest neighbor matching	0.0589	0.0139	4.24
	4-nearest neighbor matching	0.0602	0.0112	5.38
	Nearest-neighbor matching with caliper	0.0601	0.0112	5.37
	Kernel matching	0.0782	0.0099	7.89

**Table 9:** Results of the Group Difference Analysis.

Variable Name	Classification Criteria	Happiness of Migrant Worker			
		ATT	S.E.	Mean	T-Stat
Age	16–30 years old	0.0709	0.0233	3.5690	3.04
	30–45 years old	0.0877	0.0192	3.6107	4.56
	Over 45 years old	0.0675	0.0411	3.6887	1.64
Educational background	No school	0.1729	0.1484	3.5407	1.16
	Primary school	0.1057	0.0513	3.5907	2.06
	Junior middle school	0.0618	0.0220	3.5912	2.81
	Senior middle school/technical school	0.0721	0.0268	3.6135	2.69
	College	0.0919	0.0342	3.6782	2.69
	University	−0.0518	0.0429	3.7017	−1.21
	Graduate school	−0.0371	0.1906	3.6691	−0.19
Whether there is contracted land in their hometown	No	0.0524	0.0201	3.5695	2.60
	Unclear	0.0474	0.0563	3.5038	0.84
	Yes	0.0667	0.0200	3.6522	3.34
Whether they have their collective dividends	No	0.0689	0.0144	3.6151	4.79
	Unclear	0.0170	0.0586	3.5059	0.29
	Yes	−0.0873	0.0876	3.6566	−1.00
The total monthly income of their family	Under 4000 yuan	0.0738	0.0329	3.5706	2.24
	4000–6000 yuan	0.0762	0.0256	3.5968	2.97
	6000–8000 yuan	0.0594	0.0298	3.6246	2.00
	More than 8000 yuan	0.0060	0.0248	3.6553	0.24
